# Supplementary material for: Age-Dependent Sex Differences in Perineuronal Nets in an APP Mouse Model of Alzheimer’s Disease Are Brain Region-Specific
Source: Int J Mol Sci. 2023 Oct 5;24(19):14917. doi: 10.3390/ijms241914917 (PMC10574007; doi:10.3390/ijms241914917)
Supplement: Supplementary file 1 [file ijms-24-14917-s001.zip › ijms-2609615-supplementary-revised.pdf]

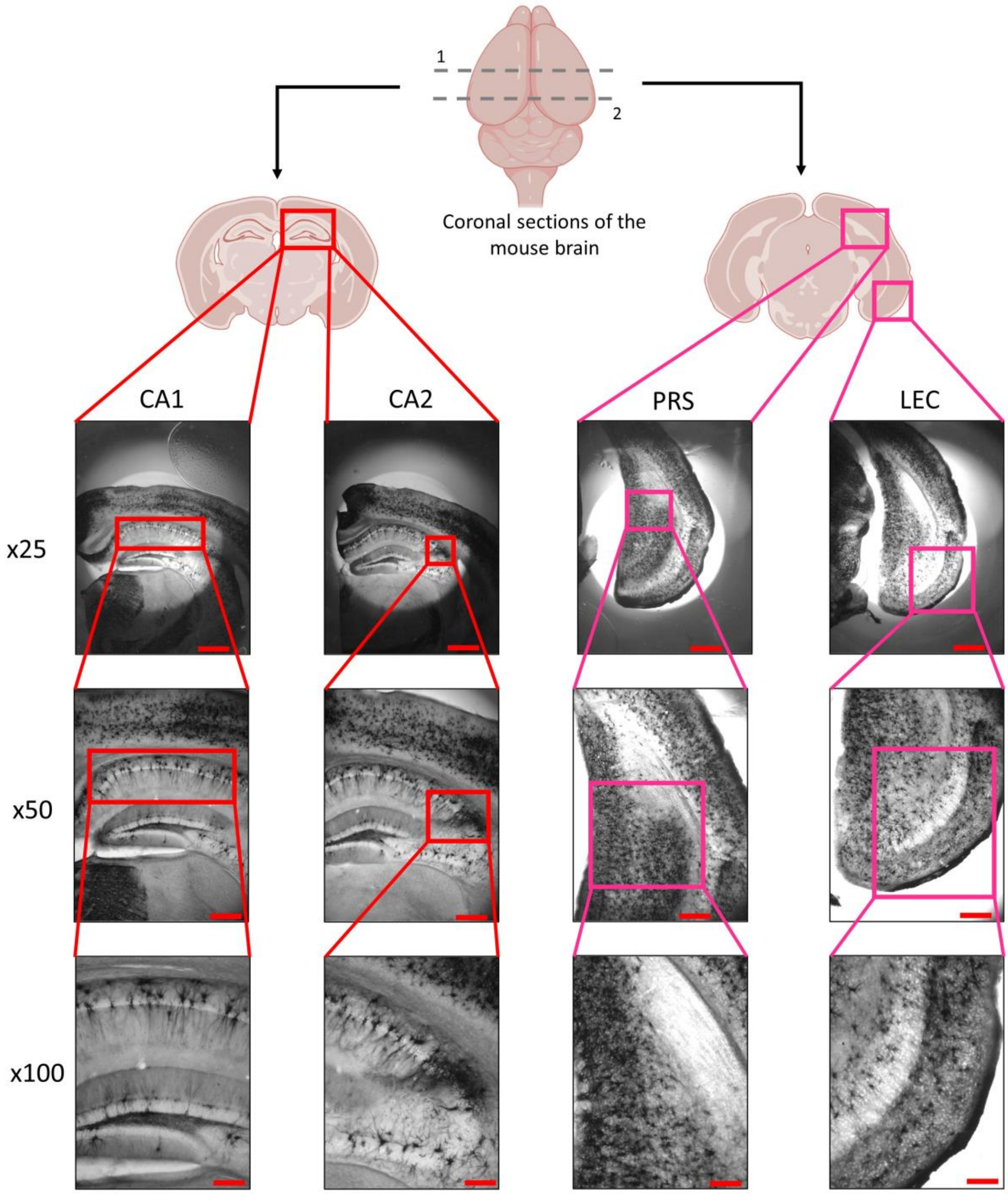

Figure S1. Representative images of whole brain section labelling of PNNs. Examples show the density of PNNs in the *APP*$^{NL\text{-}F/NL\text{-}F}$ mouse model of AD in different cortical regions. Representative brightfield images taken at x25, x50 (Scale bar 50 µm) and x100 (Scale bar 20 µm) magnification from 2-5-month-old *APP*$^{NL\text{-}F/NL\text{-}F}$ mice from CA1, CA2, LEC and PRS, respectively. Each coronal section was carefully sectioned at 100 µm thickness using a vibratome guided by the Allen mouse brain atlas to ensure consistency of brain region used for experimentation.

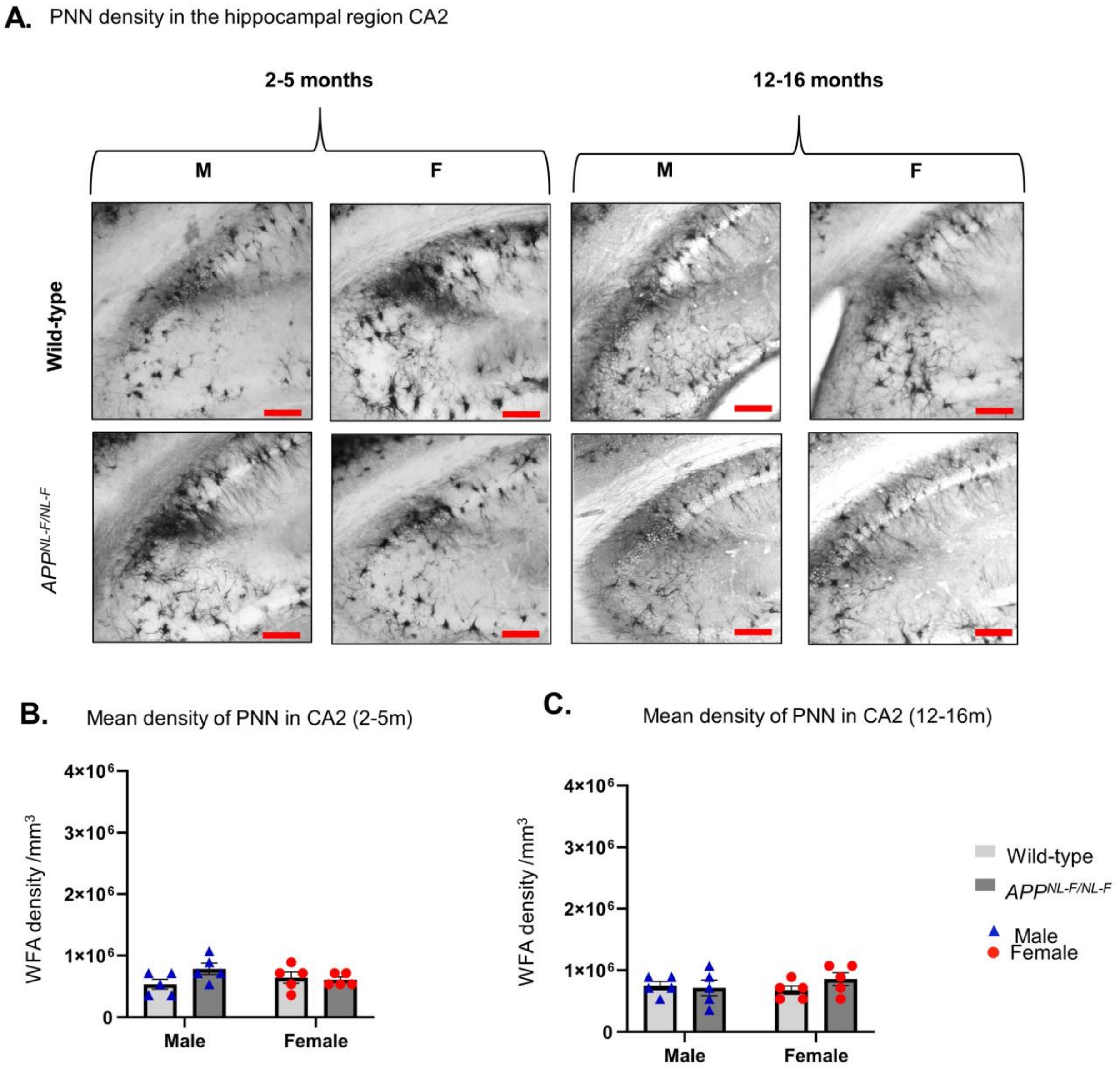

Figure S2. Alteration in the density of PNNs in the *APP*$^{NL-F/NL-F}$ mouse model of AD in CA2 region. (A) Representative brightfield images taken at x200 magnification from 2-5 month and 12-16 months old wild-type mice, age-matched to *APP*$^{NL-F/NL-F}$ mice from the CA2 hippocampal region. Scale bar 20 µm. (B-C) Graphs show the density of WFA, indicative of the PNN density in CA2 in both genotypes at both age windows; 2-5 months and 12-16 months, respectively. (Two-way ANOVA corrected for multiple comparisons with post-hoc Tukey's test, *n=5 animals per cohort*)
